# Ocular findings in neurosyphilis: a retrospective study from 2012 to 2022

**DOI:** 10.3389/fneur.2024.1472274

**Published:** 2024-11-28

**Authors:** Huanhuan Cheng, Haocheng Zhu, Gang Shen, Yaqi Cheng, Jiao Gong, Juan Deng

**Affiliations:** ^1^Department of Ophthalmology, The Third Affiliated Hospital of Sun Yat-sen University, Guangzhou, China; ^2^Department of Laboratory Medicine, The Third Affiliated Hospital of Sun Yat-sen University, Guangzhou, China

**Keywords:** neurosyphilis, pupil abnormalities, pupil light reflex (PLR), ocular syphilis, optic neuropathy

## Abstract

**Purpose:**

This study aimed to investigate ocular manifestations in patients with neurosyphilis and their association with general indexes.

**Methods:**

This retrospective study was conducted among patients who were hospitalized for neurosyphilis from 2012 to 2022. Clinical manifestations, laboratory tests, brain MRI, and ocular examinations were reviewed and analyzed.

**Results:**

A total of 106 neurosyphilis patients were included, of which 10 patients presented with ocular signs as their initial symptoms. The most common main complaint was reduced vision (6/10), followed by diplopia (2/10) and ptosis (2/10). The causes of vision loss included optic neuropathy (4/6) and posterior uveitis (2/6), with all six patients showing optic nerve involvement. A total of 29 (27.4%) patients exhibited pupil abnormalities. A lumbar puncture was performed on all 106 patients, and abnormal cerebrospinal fluid (CSF) findings were revealed in 101 (95.3%) patients, indicating central nervous involvement. The median white blood cell count in the CSF of the patients with pupil abnormalities was significantly higher than that of those without pupil abnormalities (14.0 vs. 6.0 cells/μl, *p* = 0.037). In addition, the patients with abnormal pupillary light reflex (PLR) were more likely to have multiple brain lesions compared to those with normal PLR (77.3% vs. 29.2%, *p* < 0.001).

**Conclusion:**

Optic nerve involvement is the main cause of vision loss in neurosyphilis. Patients with optic neuropathy or posterior uveitis should undergo prompt diagnostic evaluation for syphilis. Pupil abnormalities can serve as indicators of more severe CSF and MRI findings.

## Introduction

Worldwide, there is a resurgence of syphilis, which poses a serious public health concern (1). In China, the incidence of syphilis increased from 0.9 to 34.49 per million between 1990 and 2017, and the incidence of neurosyphilis showed a similar upward trend (2). Syphilis is the great masquerader due to its variable and wide spectrum of presentations. There are three stages of syphilis (primary, secondary, and tertiary), and each stage is characterized by distinct clinical signs and symptoms. *Treponema pallidum* can affect the central nervous system and lead to neurosyphilis at any stage of the disease.

Currently, there is no gold standard for the diagnosis of neurosyphilis, and the diagnosis relies on a combination of clinical and biological parameters in association with the results of serology and cerebrospinal fluid (CSF) testing (3). Examination of the CSF is considered essential in patients with syphilitic neural involvement to confirm the diagnosis of neurosyphilis and determine subsequent management (4). However, CSF testing is both invasive and time-consuming. On the other hand, brain MRI, though non-invasive, often yields protean and atypical results in cases of neurosyphilis. The eyes can serve as a window to neurosyphilis, and ocular signs may aid in its diagnosis. Previous studies have investigated the clinical characteristics of ocular syphilis and neurosyphilis patients (5). However, there is limited evidence regarding the association between ocular manifestations of neurosyphilis and other parameters, such as CSF or MRI results. In this retrospective study spanning over 10 years, we summarized the ocular findings in neurosyphilis patients and explored their association with general health conditions.

## Methods

### Study design and participants

This study was carried out with the approval of the ethical committee of the Third Affiliated Hospital of Sun Yat-sen University and adhered to the guidelines outlined in the Declaration of Helsinki. Informed consent was waived as only medical records were involved. The medical records of patients with neurosyphilis were retrospectively reviewed at the Third Affiliated Hospital of Sun Yat-sen University from September 2012 to June 2022. The inclusion criteria were as follows: patients hospitalized for neurosyphilis who were aged 18 years old or above, with available electronic medical records and retrievable testing results for syphilis from both serum and CSF from the online clinical laboratory system. Patients with other ocular or systemic diseases that might cause lesions in the posterior pole or the optic nerve, such as glaucoma, multiple sclerosis, or autoimmune diseases, were excluded.

### Data collection

Data for the final analysis included demographics, clinical manifestations, ocular symptoms, serological testing results, and MRI and CSF findings. The results of the serum and CSF analyses were obtained before intravenous penicillin treatment. Patients with ocular symptoms routinely underwent comprehensive ophthalmic examinations, including color fundus photography, optical coherence tomography (OCT), a 30–2 visual field (VF) test, and fundus fluorescein angiography (FFA) when necessary. VF severity was graded as follows: mild (≥ −3.00 dB), moderate (−3.01 to −6.00 dB), and severe (−6.01 to −20.00 dB) (6).

The results of treponema pallidum particle agglutination (TPPA) and the toluidine red unheated serum test (TRUST) were reviewed. Lumbar puncture and CSF analysis were also conducted. The CSF analysis included the number of nuclear elements, glucose, protein, and chlorine, as well as the CSF TPPA and TRUST tests. In some patients, the results of the CSF Rapid Plasma Reagin (RPR) test and FTA-ABS (fluorescent treponemal antibody-absorbed) test were also available. Normal CSF values include a cell count of <5 cells/μL, glucose levels of 45–80 mg/dL, protein levels of 0.15–0.45 g/L, and lactase levels of 10–22 mg/dL. The diagnosis of neurosyphilis was based on the neurological or ophthalmic symptoms, serological results from the blood, and biochemical analysis of the CSF, in accordance with the CDC recommendation (7).

### Statistical analysis

Due to the retrospective nature of the study, no sample size calculation was conducted. Statistical analysis was performed using SPSS 22.0 (IBM Corp., Armonk, NY, United States). Categorical variables were presented as the number (percentage) of patients. Continuous data were tested for normality using the Kolmogorov–Smirnov test or Shapiro–Wilk test. Data with a normal distribution were presented as mean ± standard deviation, while data that were not normally distributed were presented as median with IQR (P25-P75). The t-test and one-way ANOVA were used to compare the parameters with a normal distribution, while a non-parametric test was applied to the data that did not follow a normal distribution. The χ^2^ test was performed to compare the categorical variables. A *p*-value of <0.05 was considered statistically significant.

## Results

### Study population

A total of 106 neurosyphilis patients (84 male and 22 female participants) were included in the present study. The mean age was 52.3 ± 11.9 years. All patients were symptomatic, and the main initial symptoms were divided into four types: ophthalmic, neuropsychiatric, meningovascular, and myelopathic. Demographic and clinical features classified by the main symptoms are shown in Table 1. In this case series of neurosyphilis, neuropsychiatric symptoms were the most prevalent, with 27 patients exhibiting cognitive disorders and 28 presenting with altered mental status. A total of 10 (9.43%) patients exhibited ocular symptoms and underwent ophthalmic consultation during hospitalization. Lumbar puncture was performed on all patients, and abnormal CSF findings were revealed in 101 (95.3%) patients, indicating central nervous involvement. A total of 82 patients were diagnosed with definite neurosyphilis according to the CDC criteria (4). All patients tested positive for the serum TPPA test. In addition, 98 patients tested positive for the serum TRUST test, and 37 patients had a TRUST titer of >1:8.

**Table 1:** Demographics and clinical features of neurosyphilis patients classified by the main initial symptoms.

Variable	Ophthalmic	Neuropsychiatric	Meningovascular	Myelopathic	*p*-value
Number	10	55	34	7	–
Age (year)	49.5 (39.5–55.3)	50.0 (46.0–55.0)	55.0 (47.0–66.5)	52.0 (44.0–79.0)	0.087
Sex (F/M)	1/9	13/42	7/31	1/6	0.763
Normal pupil (n)	4	41	27	5	0.100
Normal pupil size (n)	7	48	31	6	0.051
Norma PLR (n)	6	41	29	5	0.026^*^
Normal MR (n)	4	10	3	3	0.100
Encephalanalosis (n)	1	22	11	3	0.21
Arteriosclerosis (n)	1	14	15	3	0.18
Serum TRUST titer>1:8(n)	3	25	6	3	0.06
CSF WBC (cells/μl)	20.5 (6.0–45.5)	6.0 (2.0–26.0)	6.0 (2.0–16.0)	8.0 (0–28.0)	0.452
CSF protein (mg/L)	0.54 (0.33–0.72)	0.50 (0.36–0.77)	0.51 (0.40–0.74)	0.55 (0.38–0.82)	0.985
CSF glucose (mg/dl)	3.53 (3.15–3.80)	3.31 (2.98–3.94)	3.37 (2.95–3.92)	3.45 (2.84–3.84)	0.964
CSF chlorine (mmol/L)	123.1 (120.2–124.5)	125.7 (123.4–128.1)	124.0 (120.9–125.3)	122.0 (120.3–125.8)	0.004^#^
CSF TRUST titer>1:8(n)	1	2	0	2	0.01^*^

PLR, pupillary light reflex; TRUST, toluidine red unheated serum test; CSF, cerebrospinal fluid; and WBC, white blood cell. *p < 0.05, #*p* < 0.01.

### Pupil abnormalities

Pupil abnormalities were documented in the medical records; however, no definite diagnosis of Adie’s pupil or Argyll Robertson pupils could be made as no near-light reflection or pilocarpine testing was performed. Of the 106 patients, 29 (27.4%) exhibited pupil abnormalities. In terms of pupil size, 14 (13.2%) patients exhibited abnormalities. Six patients had anisocoria, although the diameters of both pupils were within the normal range. Two patients presented with unilateral microcoria. Seven patients had mydriasis, with four exhibiting unilateral mydriasis and three exhibiting bilateral mydriasis. The remaining two patients had irregular pupils (one bilateral and one unilateral).

In terms of pupillary light reflex (PLR), 25 (23.6%) patients exhibited abnormalities. The most common PLR abnormality was bilateral bluntness (15/106, 14.2%), followed by bilateral disappearance (7/106, 6.6%). There was a significant difference in the distribution of the PLR types (*p* = 0.026) across the four symptom subgroups. The percentage of patients with normal PLR was lowest in the ophthalmic abnormality group (60%) and highest in the meningovascular group (85.3%).

### Ocular findings

The median duration of the ocular symptoms at the time of hospitalization was 4 months (IQR 2 to 8.75). The main ocular complaints included decreased vision in six cases, diplopia in two cases, and ptosis in two cases. The diagnoses included posterior uveitis in two patients, optic neuritis in two patients, optic nerve atrophy in two patients, oculomotor palsy in three patients, and abducens palsy in one patient. Bilateral involvement occurred in three patients (Table 2).

**Table 2:** Demographic data, serological status, cerebrospinal fluid, OCT, and visual field findings of neurosyphilis patients with ocular symptoms as the initial presentation.

Case	Age/Sex	Ocular symptom	Ocular sign	Ocular examination	Ophthalmic diagnosis	Serological	CSF
Case 1	57/F	Reduced vision	Unilateral PLR disappearance	OCT: pRNFL thinning OU	Posterior optic neuritis	TPPA>1:1280 TRUST1:1	TPPA 1:160
Case 2	38/M	Diplopia	Moderate limited abduction OD	Normal OCT findings, VF: borderline OD, paracentral scotoma in the nasoinferior quadrant OS	Abducens palsy	TPPA+ TRUST1:2	FAT-ABS+ TPPA 1:1280
Case 3	50/M	Ptosis& diplopia	Restricted eye movement OD	VF: multiple non-systematic defects in the 4 quadrants OD, general reduction of sensitivity OS	Oculomotor palsy	TPPA+ TRUST1:2	TPPA+ TRUST+
Case 4	64/M	Ptosis	Ptosis	Unremarkable	Oculomotor palsy	TPPA+ TRUST+	TPPA+ TRUST+
Case 5	53/M	Diplopia	Asymmetrical pupils	Unremarkable	Oculomotor palsy	TPPA+ TRUST- due to dilution	TPPA+ TRUST- due to dilution
Case 6	29/M	Reduced vision OU	Papilledema, retinal vasculitis OU	FFA: dilated retinal vessels with leakage; diffuse telangiectases and leakage of optic disk OU VEP: decreased amplitudes of all waves OU	Posterior uveitis	TPPA+ TRUST1:64	TPPA+ TRUST1:64
Case 7	49/M	Reduced vision	Unilateral pale optic disk	Gradual VF constriction from periphery	Optic nerve atrophy	TPPA+	FTA-ABS+ TPPA>1:1280 TRUST1:2 RPR1:4
Case 8	62/M	Reduced vision	Unilateral pale optic disk	–	Optic nerve atrophy	TPPA+ TRUST1:8	TPPA+ TRUST+
Case 9	40/M	Reduced vision OU, rotation pain OS	Bilateral dull light reflection	Unremarkable	Posterior optic neuritis	TRUST1:64 TPPA>1:1280 RPR 1:128	FTA-ABS+ VDRL1:8 RPR 1:8
Case 10	45/M	Reduced vision OS		VF: central scotoma OD; FFA: retinal vein dilation and leakage, ill-demarcated optic disk border at late stage, widow defect of RPE.	Posterior uveitis	TPPA 1:1280 TRUST 1:64	TPPA+ TRUST- due to dilution

NA, not available; OD, oculus dexter; OS, oculus sinister; OU, oculus uterque; FFA, fluorescein angiography; VF, visual field; RPE, retinal pigment epithelium; VEP, visual evoked potential; OCT, optical coherence tomography; CSF, cerebrospinal fluid; TPPA, treponema pallidum particle agglutination test; RPR, rapid plasma reagin test; and TRUST, toluidine red unheated serum test.

**Figure 1:**
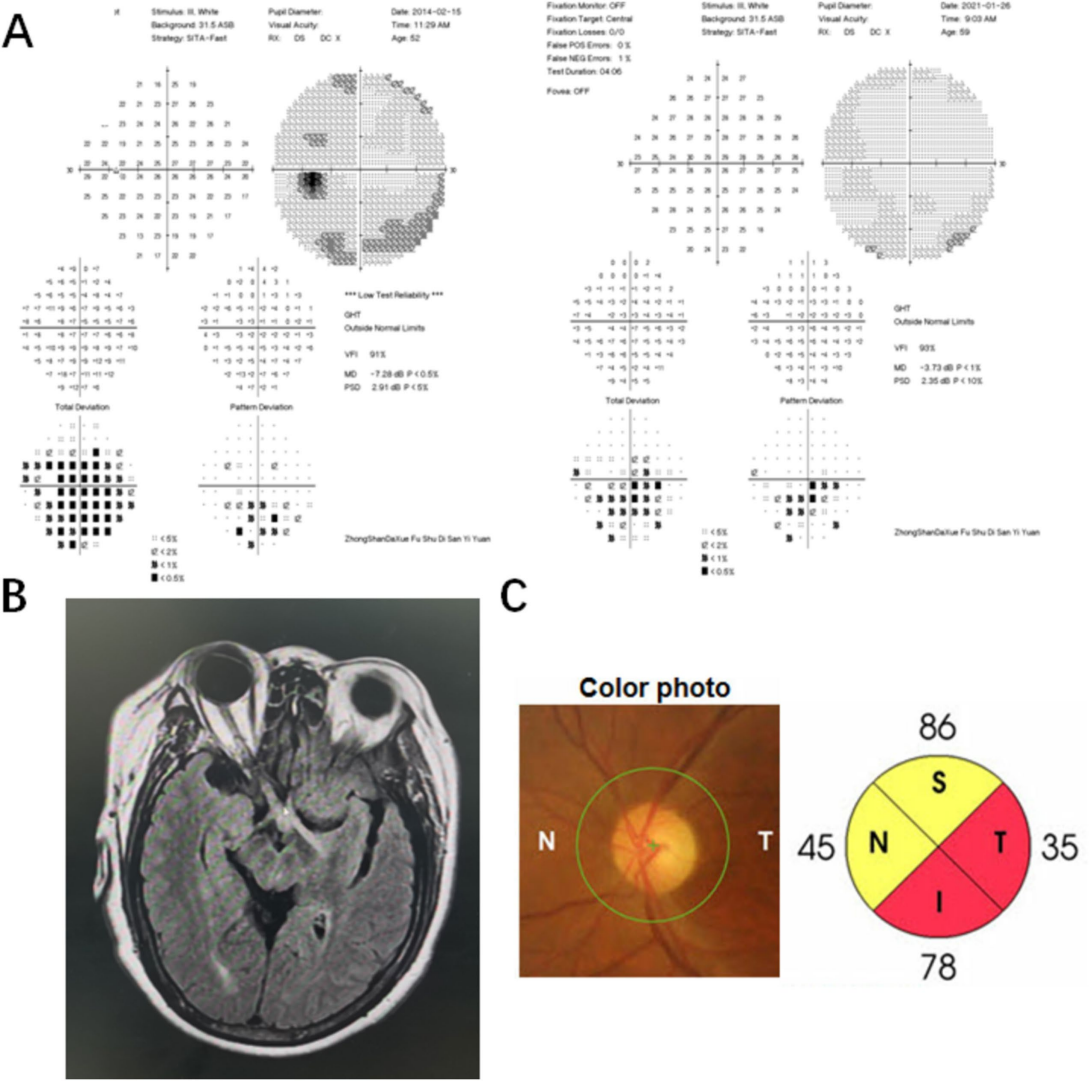
Visual field (VF), MRI, and optical coherence tomography (OCT) results of a neurosyphilis patient presented with posterior optic neuritis. (A) The VF result showed a dense inferior altitudinal defect in the left eye. The MD improved from severe damage (−7.28 dB) to moderate damage (−3.73 dB) in the left eye after standard penicillin treatment; (B) The MRI result revealed increased signaling of the optic nerve and optic chiasm on the right side, bilateral optic tract lesions, minor degeneration of the right frontal lobe and left parietal lobe upon hospitalization; (C) The fundoscopy showed the optic disk with normal morphology in the left eye. Temporal and inferior peripapillary retinal nerve fiber layer atrophy in the left eye persisted over a 7-year follow-up period.

Case 1 involved a patient who experienced optic neuritis in the right eye 8 years ago. The patient received steroid treatment elsewhere and went through periods of recovery and relapse. The patient was hospitalized due to sudden blurred vision in the left eye, which persisted for 2 months, and the visual acuity was limited to light perception in the right eye and measured at 20/200 in the left eye. The VF test revealed a dense inferior altitudinal defect in the left eye, while the vision in the right eye was too poor to conduct the test. Testing for AQP4 and MOG antibodies was negative. After confirming the diagnosis of neurosyphilis, standard penicillin treatment was carried out. MD improved from severe damage (−7.28 dB) to moderate damage (−3.73 dB), but temporal and inferior peripapillary retinal nerve fiber layer atrophy persisted over a 7-year follow-up period. (Figure 1). Case 3 was a typical example of secondary ophthalmoplegia. The patient complained of left eye ptosis for 2 months and right eye ptosis for 20 days, accompanied by diplopia. Prominently restricted eye movement was observed in the right eye. An MRI revealed meningitis, suggesting that oculomotor paralysis was due to syphilitic meningitis.

Of the two patients with syphilitic posterior uveitis, the FFA demonstrated prominently dilated retinal vessels with associated leakage. In case 6, diffuse telangiectases and leakage corresponding to optic disk elevation and hyperemia were observed in both eyes, with no obvious obstruction of the retinal vessels. No raised CSF pressure was documented. The Brain MRI was normal. Visual evoked potentials revealed decreased amplitude of all waves in both eyes. The patient was diagnosed with bilateral neuroretinitis. In case 10, retinal vein dilation and leakage, as well as telangiectases, mainly affected the posterior, inferior, and temporal-inferior mid-peripheral retina in the left eye. Telangiectases over the optic disk with moderate leakage and an ill-demarcated border at the late stage were also observed in the left eye. In addition, the FFA showed needle-point-shaped hyperfluorescence in the macula and inferior mid-periphery. The VF test revealed a central scotoma in the right eye. The patient was diagnosed with acute syphilitic posterior placoid chorioretinopathy (ASPPC) in the left eye. Other infectious or granulomatous causes were ruled out in the patients who initially presented with posterior uveitis. Of the six patients with decreased vision, five showed no improvement in VA. No other ocular features, such as gummas iridocyclitis, or retinal vascular occlusions, were present in the 10 patients who initially presented with isolated ocular involvement.

All 10 patients were reactive for the serum TPPA and TRUST. In the CSF analyses, the white blood cell count exceeded 5× 10^6^/L in eight patients, and the protein level reached 0.5 g/L or higher in six patients. All patients were also reactive for the CSF TPPA and TRUST.

### Serological and CSF findings

All patients underwent testing for the serum TPPA and TRUST. As is shown in Table 1, among patients with ophthalmic abnormalities, three had a serum TRUST titer over 1:8, while one patient had a CSF TRUST titer over 1:8. The median CSF WBC count was 20.5 cells/μl (IQR 6.0–45.5 cells/μl). The concentrations of protein and glucose in the CSF were 0.54 mg/L (IQR 0.33–0.72 mg/L) and 3.53 mg/dL (IQR 3.15–3.80 mg/dL), respectively. The CSF chlorine level was 123.1 mmol/L (IQR 120.2–124.5 mmol/L). The CSF chlorine level in neuropsychiatric patients was significantly higher compared to those with meningovascular symptoms (*p* = 0.022).

The patients with neuropsychiatric symptoms had the highest proportion of a serum TRUST titer over 1:8 (45.5%), while the patients with meningovascular neurosyphilis had the lowest proportion (17.6%). The difference between the groups was not significant, although it approached statistical significance (*p* = 0.06). The patients with myelopathic symptoms initially had a significantly higher proportion of a CSF TRUST titer over 1:8 (28.6%) compared to the patients in any other symptom groups (*p* = 0.01). No significant difference was found between the symptom subgroups in terms of other CSF parameters.

**Figure 2:**
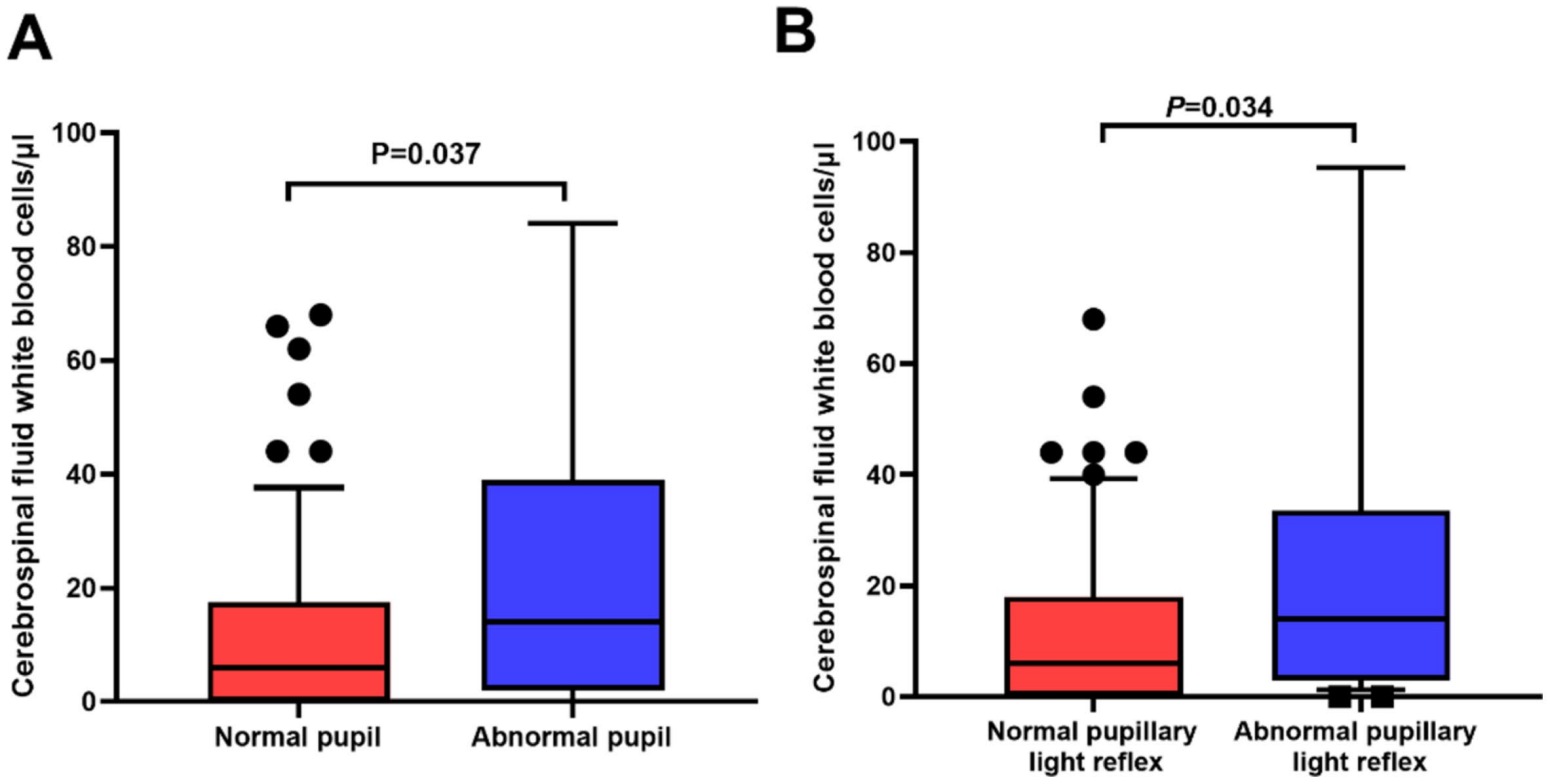
Significant differences in cerebrospinal fluid (CSF) white blood cell counts in relation to pupil abnormalities in neurosyphilis patients. (A) The patients with abnormal pupils had higher CSF white blood cell counts than those with normal pupils (*p* = 0.037); (B) The patients with abnormal pupil light reflex (PLR) had higher CSF white blood cell counts than those with normal PLR (*p* = 0.034).

CSF metrics in relation to pupil abnormalities were also investigated. The median CSF WBC count in the patients with pupil abnormalities was significantly higher compared to those without pupil abnormalities (14.0 vs. 6.0 cells/μl, *p* = 0.037). In addition, the patients with abnormal PLR had higher CSF WBC counts than those without abnormal (*p* = 0.034; Figure 2). No significant difference was found when comparing other CSF parameters, such as the concentrations of protein, sugar, or chlorine.

### MRI findings

A total of 94 neurosyphilis patients underwent brain MRI examinations. The MRI findings were divided into five categories: normal, frontal lobe degeneration, frontal–parietal lobe degeneration, multiple degenerations involving the frontal–parietal lobe, and degeneration involving areas other than the frontal–parietal lobe. The most common type was multiple degenerations involving the frontal–parietal lobe (42/94, 44.7%), and 20 patients had normal MRI results. The patients with pupil abnormalities tended to have multiple brain lesions compared to those without pupil abnormalities (76.0% vs. 27.5%, *p* < 0.001). Further analysis revealed that the patients with abnormal PLR were more likely to have multiple brain lesions than those with normal PLR (77.3% vs. 29.2%, *p* < 0.001). However, no significant difference was found regarding abnormalities in pupil size (61.5% vs. 37.0%, *p* = 0.095).

Regarding the brain MRI in the patients with ophthalmic abnormalities, two patients refused the examination, two had normal results, and three had multiple degenerative lesions involving the frontal lobe and parietal lobe. In case 1, inflammation of the optic nerve and optic chiasm on the right side was noted, along with bilateral optic tract lesions, minor degeneration of the right frontal lobe and the left parietal lobe, and cerebral arteriosclerosis. In case 3, thickened and intensified endocranium signaling was also observed, suggesting meningitis.

## Discussion

Neurosyphilis is a relatively rare disease; however, it can lead to serious consequences. In this study, 9.43% of neurosyphilis patients presented with ocular signs as their initial primary complaint and 5.66% of the patients had ocular syphilis. Pupil abnormalities were common in neurosyphilis patients (29/106, 27.4%). In addition, we found for the first time that abnormal PLR served as an indicator of multiple intracranial lesions and a higher WBC count in the CSF.

In a previous study, ocular signs led to the diagnosis of syphilis in up to 78% of patients (8). Other studies have reported varying incidences of ocular manifestations in syphilis. The incidence of ocular symptoms in syphilis patients was reported to be 0.6% in the United Kingdom (9), while 0.65% of patients in the United States had comorbid suspected ocular syphilis (10). In eastern China, ocular syphilis was comorbid in 5.1% of syphilis cases (11), which was similar to the rate reported in this southern Chinese population with neurosyphilis.

Among syphilis patients exhibiting diplopia, the majority presented with oculomotor palsy (12), a finding also reflected in this study, where three cases had oculomotor palsy (cases 3, 4, and 5). In case 3, oculomotor palsy, which presented as binocular diplopia and ptosis, was secondary to syphilitic meningitis. The sudden onset of binocular diplopia in case 5, although not specific, was more suggestive of vascular causes (13), which was later confirmed by MRA. Due to the lack of systemic ocular screening among all the neurosyphilis patients in this study, there may be more cases of ocular involvement than currently reported.

Ocular syphilis mainly presents as posterior uveitis, affecting the choroid, retina, and optic nerve, and often occurs simultaneously with neurosyphilis (14). Typical signs of syphilitic uveitis, including ASPPC, retinal necrosis, and punctate inner retinopathy, were less frequent in this case series. In contrast, optic nerve involvement was observed in all six patients. Syphilitic optic neuropathy can present as perineuritis, retrobulbar optic neuritis, or optic neuritis with disk swelling (papillitis) (15). Papillitis and posterior placoid chorioretinopathy can exist simultaneously in a syphilis patient (16). In this study, papillitis and retinal vasculitis existed simultaneously in one patient (case 6). The diagnosis of eye involvement in syphilis may be delayed due to the lack of specificity, low suspicion for syphilis, fluctuating symptoms, and similarities in presentation to other diseases (17). Timely treatment with penicillin within 28 days of symptom onset is crucial for prognosis as delayed diagnosis and treatment of ocular syphilis may result in permanent visual impairment (18). Unfortunately, in this study, the minimum duration of ocular symptoms was 2 months. Two patients received vitamin B12 or steroid treatment elsewhere, and two patients had already developed optic nerve atrophy before penicillin treatment, which explained the poor visual outcome. Steroids are an important adjunct to the treatment of syphilitic uveitis and optic neuritis (19). Of the six patients with syphilitic optic neuropathy and posterior uveitis in this study, only two were treated with systemic steroids. A combined treatment of steroids with penicillin might have facilitated better visual recovery than what was reported in the described cases.

MRI abnormalities in neurosyphilis are protean and mimic many other neurological disorders (20). The most common MRI diagnostic features were reported to be medium contrast enhancement, atrophy, white matter lesions, cerebral infarction, and edema (21). Brainstem and thalamic lesions can lead to pupil abnormalities. However, in this study, only two patients had thalamic lesions and no patient had brainstem involvement.

In the present study, among the 94 patients with MRI results, 66 patients (70.2%) had frontal lobe involvement, 45 patients (47.9%) had parietal lobe involvement, and 18 patients (19.1%) had temporal lobe involvement, which explained the dominant neuropsychiatric symptoms. Similarly, in a previous study on the characteristics of patients with general paresis of the insane, which mainly manifests as cognitive disorders and neuropsychiatric symptoms, abnormal signals were mostly distributed in the frontal lobe and temporal lobe (22).

Previous studies have investigated the potential of PLR as a diagnostic tool (23). PLR is mediated by melanopsin-based photoreception, which activates a cerebral network, including frontal regions that are classically involved in attention and ocular motor responses (24). In line with previous studies, we found that the majority of neurosyphilis patients (70.2%) had frontal lobe lesions. The diagnosis of neurosyphilis remains challenging, and a CSF cell count of more than five leukocytes is supportive of the diagnosis (25). Abnormal PLR was associated with a higher leukocyte count in the CSF, which may indicate more severe cerebral neuroinflammation. Our results suggest that neurosyphilis should be taken into consideration when encountering frontal lobe involvement accompanied by abnormal PLR.

This study has several limitations. First, because of the retrospective nature of the study, data were retrieved from medical records, which only recorded the status of pupil size, shape, and light reflex. Asymmetric, irregular, and miotic pupils with light-near dissociation, namely Argyll Robertson pupils, are a prominent feature of neurosyphilis. However, in this study, no more detailed examination of the pupils, such as near-light reflection or pilocarpine testing, was available. Therefore, it was challenging to determine the presence of Adie’s pupil or Argyll Robertson pupils and investigate its association with other parameters. In addition, the swinging flashlight test is subjective, and objective results obtained using pupillometry may yield more convincing conclusions. Further prospective studies with more meticulous examinations of the pupils are warranted. Second, no HIV-positive patients were included in this study. The relationship between HIV infection and syphilis is well-established, and HIV may increase the likelihood of syphilis progressing into neurosyphilis (4). The clinical features of patients with both syphilis and HIV infection were not presented in this study. Third, because of the limited sample size of the subgroups, some comparisons did not yield statistically significant results. A future study with a larger sample size may reveal more prominent findings. In addition, due to the retrospective nature of this study, many patients were lost to follow-up after hospitalization and few had serological retesting for syphilis. As a result, this study was underpowered to detect clinical differences in treatment response to penicillin between the symptom subgroups. The strengths of the present study include a reasonably large sample size of neurosyphilis patients and a focused emphasis on ocular manifestations and their association with general health conditions.

Overall, the incidence of ocular abnormalities as the initial symptom of neurosyphilis is 9.43% (10/106) in this study. Optic nerve involvement is the main cause of vision loss in neurosyphilis. Patients with optic neuropathy or posterior uveitis should be evaluated for syphilis, as early treatment is crucial for preventing irreversible vision loss. Pupil abnormalities and abnormal PLR can serve as indicators of positive CSF and MRI findings.

## Data Availability

The raw data supporting the conclusions of this article will be made available by the corresponding author upon request.
